# Bone age assessment: comparative analysis of Greulich-Pyle and Tanner-Whitehouse 3 by pediatric radiologists and endocrinologists

**DOI:** 10.1186/s12880-026-02350-y

**Published:** 2026-04-18

**Authors:** Nipaporn Tewattanarat, Wichuda Chaisiwamongkol, Pitchaya Wiratchotisatian, Sarun Paisarnsrisomsuk, Panawit Hanpinitsak, Kwankhao Tangprasert, Chanakarn Poonpol, Chatparin Pansukrada, Wilairat Thaowandee, Ratikorn Chaisiwamongkol

**Affiliations:** 1 Department of Radiology, Faculty of Medicine, Khon Kaen University, Khon Kaen, Thailand; 2 Department of Statistics, Faculty of Science, Khon Kaen University, Khon Kaen, Thailand; 3 Department of Computer Engineering, Faculty of Engineering, Khon Kaen University, Khon Kaen, Thailand; 4 Information Technology Workgroups, Faculty of Science, Khon Kaen University, Khon Kaen, Thailand; 5 Department of Pediatrics, Faculty of Medicine, Khon Kaen University, 123 Mittraphap Road, Mueang, Khon Kaen, 40002 Thailand

**Keywords:** Bone age assessment, Greulich and Pyle, Tanner and Whitehouse, Reproducibility, Children

## Abstract

**Introduction:**

Two widely used bone age assessment (BAA) methods**—**Greulich-Pyle (GP) and Tanner-Whitehouse 3 (TW3) methods—differ in complexity, accuracy, and clinical utility.

**Objective:**

To compare GP and TW3 bone age estimations and assess inter- and intra-observer agreement between pediatric radiologists and endocrinologists.

**Methods:**

This retrospective study analyzed 1,725 left-hand radiographs of children aged 0–19 years (2008–2022). Twelve experts (six radiologists, six endocrinologists) independently assessed bone age using GP and TW3 radius-ulnar-short bones (RUS) methods following standardized training. Assessment time and method preference were recorded. Statistical analyses included descriptive statistics, intra-class correlation coefficients (ICC), Bland–Altman analysis, and comparative tests (*p* < 0.05).

**Results:**

GP estimates ranged from 6 to 228 months, whereas TW3-RUS estimates were slightly lower due to age-range limitations. Inter- and intra-observer reliability were excellent for both methods (ICC > 0.9). Mean bone age did not differ significantly between specialties using GP, whereas minor differences were observed with TW3-RUS for overall (*p <* 0.001) and when stratified by sex (males *p =* 0.016, female *p* = 0.005). Age-stratified analysis demonstrated low mean absolute differences (MAD) between specialties across most age groups, with slightly greater variability in 84.1–180 months. MAD between GP and TW3-RUS was modest in most age groups (approximately 2.5–3.3 months) but increased in older adolescents, particularly ≥ 180 months. GP assessments were significantly faster than TW3-RUS (*p* = 0.002). Experts preferred GP for routine use due to speed and ease, while TW3-RUS offered greater accuracy, specificity, and detail for complex and borderline cases.

**Conclusions:**

Both specialists demonstrated excellent agreement using both GP and TW3-RUS. We recommend GP as the primary method, reserving TW3-RUS for complex cases. Moreover, developing a GP-analog atlas with narrower, finely defined age intervals may improve clinical applicability.

## Introduction

Bone age assessment (BAA) is a medical method used to evaluate skeletal maturity in children and adolescents. It provides essential information for a wide range of clinical applications, including the diagnosis of growth and puberty disorders, monitoring the effectiveness of therapy, evaluating pubertal timing, predicting adult height and managing patients with growth and puberty related endocrine conditions [1]. Beyond endocrinology, BAA also plays a role in orthopedics, and forensic medicine, where accurate estimation of biological age has implications for both patient management and medicolegal decision-making [2, 3].

Various imaging modalities, including radiograph, ultrasound, computed tomography (CT) and magnetic resonance imaging (MRI), are employed to assess bone age by evaluating different skeletal sites such as the hand and wrist, elbow, teeth, clavicle, iliac bone, and knee [4]. Each modality has unique advantages and limitations: ultrasound and MRI offer radiation-free alternatives, while CT provides high spatial resolution for detailed bone evaluation. Among these, radiograph of the non-dominant hand and wrist remains the most widely used imaging modality for BAA as the sequence of ossification in this region follows a relatively predictable pattern and is well represented in existing reference standards [5]. In clinical practice, BAA involves comparing the patient’s radiograph with reference images in established atlases. Two main interpretation approaches are the Greulich-Pyle (GP) method and Tanner-Whitehouse (TW) method [6, 7]. The GP method, favored for its simplicity and speed, is widely utilized by radiologists and pediatricians. However, it has inherent limitations, including broader age intervals and lack of reference images for certain ages, leading to inter-observer variability and reduced precision, particularly in age groups with limited reference images [1, 8]. In contrast, the TW method systematically scores individual bones to generate a composite maturity score, offering a more detailed and reproducible assessment, albeit at the expense of increased time and complexity.

Given these differences, there remains an important question about how closely these two methods align in practice and how the level of training and specialty of the reader influences diagnostic consistency. Therefore, the present study aims to evaluate the agreement and differences in BAA using the hand and wrist radiographs by comparing the GP and TW3 methods, as well as to assess inter-specialist concordance between pediatric radiologists and pediatric endocrinologists.

## Methods

### Data sources

This study was approved by the institution’s research ethic board (protocol number HE674010). The requirement for informed consent from patients and their families was waived due to the retrospective nature of the study.

Left-hand radiographs obtained for BAA in children aged 0–19 years between September 2008 and December 2022 were retrospectively collected from patients evaluated at our tertiary-care university hospital. All patients were of Thai (Southeast Asian) ethnicity. Bone age examinations were performed for clinical indications including suspected precocious puberty, delayed puberty, abnormal growth patterns (short or tall stature), and other suspected endocrinological disorders. Radiographs were excluded if they exhibited suboptimal image quality, improper positioning, or structural bony abnormalities. Cases involving patients with confirmed genetic syndromes or skeletal dysplasia were also excluded to minimize potential confounding factors affecting bone age assessment. A total of 1,725 radiographs were included, each obtained from a distinct individual patient. The cohort comprised cases both concordant and discordant with chronological age. Concordance was defined as a bone age within *±* 2 standard deviation (SD) of chronological age, whereas discordance was defined as a deviation exceeding *±* 2 SD [6]. Among the included cases, 1,015 radiographs were concordant with chronological age (430 males and 585 females), whereas 710 radiographs were discordant (375 males and 335 females). Bone age radiographs were acquired under standardized protocols using a posteroanterior (PA) projection of the left hand and wrist, with the hand flat and fingers slightly separated. Exposure was tailored to patient size and age (typically 45–50 kVp, 2–3 mAs, and SID 100 cm).

All radiographs were retrieved from our hospital picture archiving and communication system (GE Centricity PACS Radiology RA100 Workstation; GE Healthcare, Barrington, IL).

### Image analysis

Left-hand radiographs were reviewed by twelve expert physicians, comprising six pediatric radiologists and six pediatric endocrinologists. The pediatric radiologists had between 6 and 30 years of experience in BAA (median 10.0 years, mean 12.5 years (SD 8.71), while the pediatric endocrinologists had between 3 and 10 years of experience (median 4.5 years, mean 6.0 years, SD 3.16). All experts were proficient in the GP method; however, only two pediatric radiologists and three pediatric endocrinologists had prior practiced the TW3 method. Therefore, before reviewing the radiographs, a tutorial session was conducted by a pediatric radiologist experienced in BAA using both the GP and TW3 methods.

The physicians were divided into four groups of three, with each group including both radiologists and pediatricians. The group assignment was performed randomly. Each group received 431 or 432 images for review. Patient identifying information, except sex, was concealed for the interpretation.

**Fig. 1:**
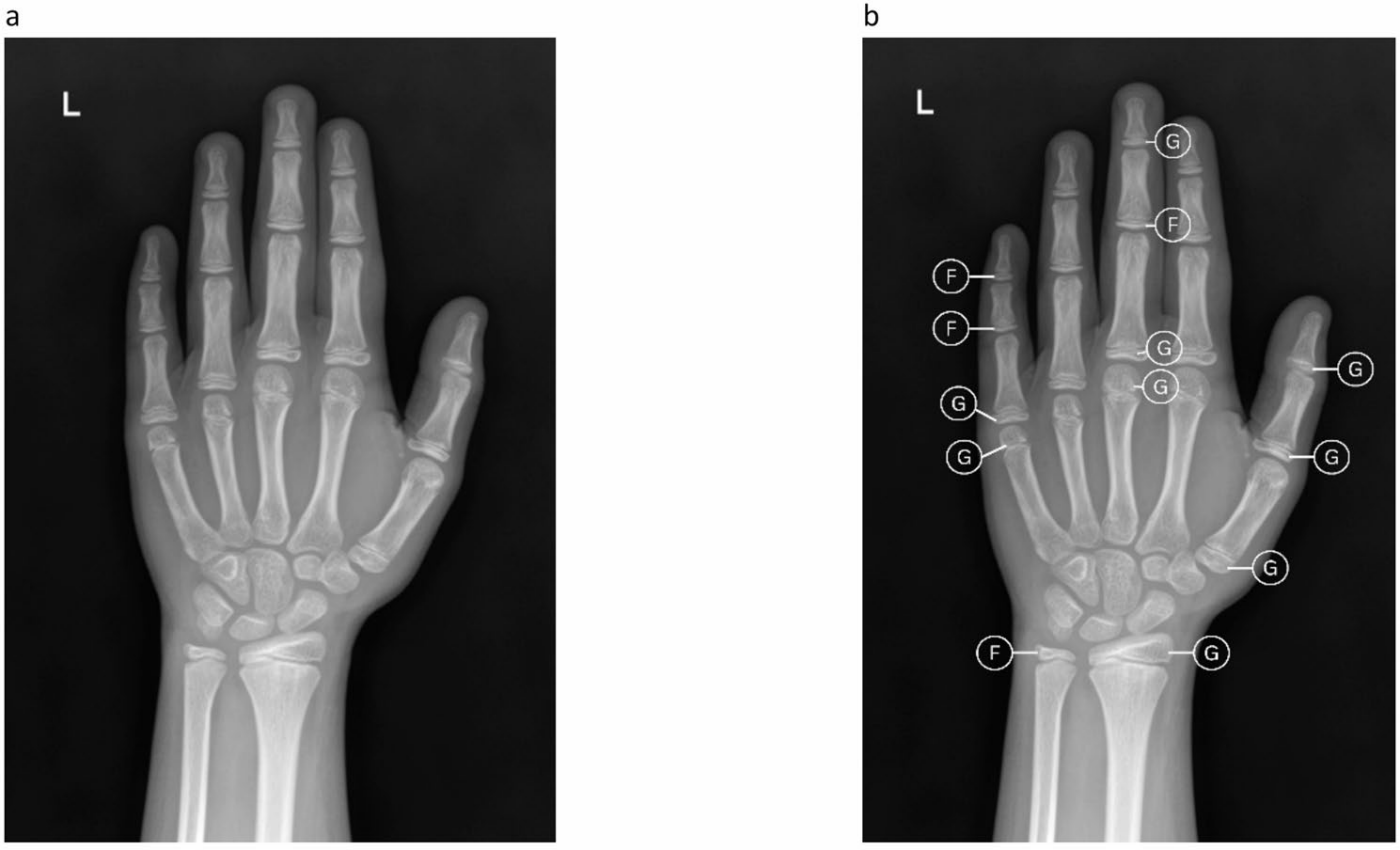
Hand radiograph of a female child. (**a**) The GP method matched the radiograph to the 11-year atlas standard. (**b**) TW3-RUS yielded a bone age of 10 years 9 months based on individual bone staging and cumulative scoring

Each expert independently and blindly evaluated the radiographs, recording the results in Google Sheets. For each image, bone age was assessed using both the GP and TW3 methods during the same session; the order of interpretation depended on the individual reader’s routine practice. Each method was completed and recorded before assigning a final estimated bone age. “Expert opinion” was defined as the final bone age determined by each reader after completing both the GP and TW3 radius-ulnar-short bones (RUS) evaluations, reflecting the reader’s clinical experience and overall judgment. All results were reported in years and months. For the GP method, each radiograph was compared to the closest matching reference images in a standard atlas [6]. For the TW3-RUS method, each of 13 bones—including the radius; ulna; 1st, 3rd, 5th metacarpals (MCP); 1st, 3rd, 5th proximal phalanges (PP), 3rd, 5th middle phalanges (MP); and 1st, 3rd, 5th distal phalanges (DP)—was individually scored based on corresponding reference images [7]. The total RUS score was categorized from A to I and subsequently converted to bone age through automated computation (Fig. 1).

**Fig. 2:**
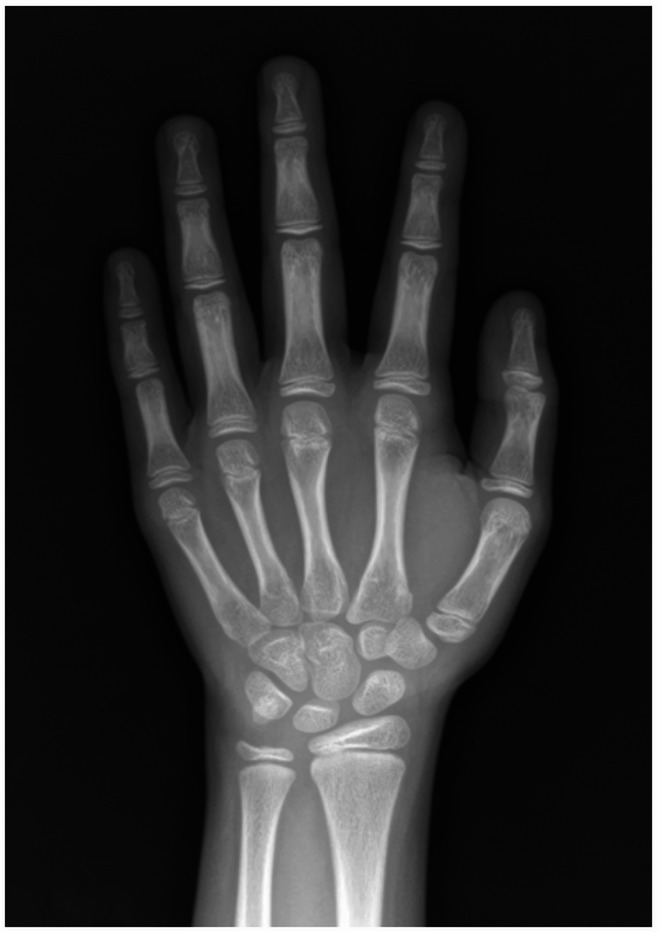
Inter-reader discrepancy in a hand radiograph of a female child. GP estimates among readers were 8 years 10 months, 11 years 0 months, and 10 years 0 months, whereas TW3-RUS estimates were 9 years 8 months, 10 years 0 months, and 10 years 0 months

Within each assessment group, consistency required agreement within six months between any two of the three readers; otherwise, they were classified as a discrepancy (Fig. 2). Discrepancies—primarily attributed to inter-reader interpretative variability, incomplete data entry, or technical recording errors—underwent second- and third-round blinded independent reassessments, within a five-month washout period between the first and second rounds and a three-week interval before the third round. The third round served to minimize residual disagreement. Any remaining discrepancies after the third round were resolved by a within-group expert consensus to establish the final reference bone age for analysis.

After completing the BAA, the experts filled out a structured questionnaire about the evaluation process. The questionnaire consisted of three sections: respondent demographics, estimated assessment duration (per case), and opinions on interpreting hand radiographs for bone age.

### Statistical analysis

Descriptive statistics (percentages, means with SD, and medians with ranges) were used to summarize patient demographics, respondent characteristics, opinions and mean absolute difference (MAD) values.

Inter-rater agreement, including comparisons between the two specialist groups and among three evaluators, was assessed using intra-class correlation coefficients (ICC) derived from a two-way mixed-effects model for absolute agreement, treating raters as fixed effects. Both single-measure (ICC [3,1]) and average-measure (ICC [3,2] and ICC [3,3]) estimates were reported, with single-measure ICC used as the primary metric to reflect agreement at the individual rater level. A 95% confidence interval (CI) was calculated for all ICC estimates. For inter-observer agreement, the ICC was calculated prior to the expert consensus process using a verified dataset that included: (1) cases with inter-reader agreement in the first round; (2) discrepant cases in the first round that were not attributable to incomplete data-entry or technical recording errors; and (3) cases initially discrepant due to incomplete data entry or technical recording errors that were subsequently corrected through a second round of blinded independent reassessment. Intra-rater reliability was evaluated in cases with repeated evaluation by the same reader, comparing the first and second readings as well as the second and third readings using the same ICC model.

Associations between bone age estimates obtained by different methods and readers were evaluated using Pearson correlation coefficients, with Spearman’s rank correlation coefficients calculated as sensitivity analyses to confirm associations without assuming linearity. ICC values were interpreted as follows: < 0.50, poor; 0.50–0.75, moderate; 0.75–0.90, good; and > 0.90, excellent [9]. Agreement between methods was further examined using Bland-Altman analysis. The magnitude of disagreement was quantified using MAD with SD.

Differences in mean bone age estimates were analyzed using paired samples *t*-tests and repeated-measures ANOVA with Greenhouse-Geisser correction, as appropriate. Normality of paired differences was assessed using Shapiro-Wilk and Kolmogorov-Smirnov tests. Despite deviations from normality, parametric tests were applied given their robustness in large samples. Non-parametric sensitivity analyses were performed using Wilcoxon signed-rank and Friedman tests.

To evaluate the influence of reader experience, MAD between each reader’s bone age estimate and the reference bone age was analyzed across three experience groups (< 6 years, 6–9 years, and > 9 years). Group differences were assessed using one-way ANOVA with Levene’s test for homogeneity of variances and Tukey HSD for post hoc comparisons. The Kruskal-Wallis test was additionally performed as a non-parametric analysis.

Responses to open-ended questions were analyzed qualitatively using content analysis. Statistical significance was set at *p* < 0.05.

All analyses were performed using IBM SPSS Statistics, version 28 (IBM Corp., Armonk, NY, USA).

## Results

A total of 1,725 radiographs were analyzed, comprising 920 from females (53.3%) and 805 from males (46.7%). When classified by chronological age, the largest proportion of cases was in the 84.1–132 months (39.6%), followed by the 132.1–180 months group (27.2%). The youngest age group (0–36 months) and the oldest group (*≥*216.1 months) comprised relatively small proportions of the study population (Table 1). One radiograph from a very young girl could not be assigned a bone age, as it did not conform to the reference criteria of either method, and was excluded from the analyses. Consequently, 1,724 radiographs were utilized for the baseline analysis.

**Table 1:** Distribution of radiographs by sex and age group

Sex	Chronological age groups (months)						Total
	0–36	36.1–84	84.1–132	132.1–180	180.1–216	≥216.1	
**Males** (no. (%))	18 (2.2)	167 (20.7)	238 (29.6)	260 (32.3)	116 (14.4)	6 (0.7)	805 (100)
**Females** (no. (%))	27 (2.9)	173 (18.8)	445 (48.4)	210 (22.8)	59 (6.4)	6 (0.7)	920 (100)
**Total** (no. (%))	45 (2.6)	340 (19.7)	683 (39.6)	470 (27.2)	175 (10.1)	12 (0.7)	1,725 (100)

*no.*, number

Bone ages, as determined by the GP method and expert opinion, ranged from 6 to 228 months (up to 19 years), consistent with the upper limit of the atlas reference range. In contrast, the TW3-RUS method produced lower estimates, ranging from 25 months (2.1 years) to 198 months (16.5 years) reflecting its inherent scoring limitations, with a lower limit threshold of 2 years and an upper threshold of 15 years for females and 16.5 years for males. Radiographs yielding bone age estimates beyond the TW3 scoring range were excluded from the TW3 analysis.

### Agreement in hand bone age assessment

Inter-rater reliability among all evaluators and inter-specialty reliability between pediatric radiologists and pediatric endocrinologists demonstrated excellent agreement across all BAA methods (Table 2). Among all evaluators, the single-measure ICC values were 0.980 (95% CI: 0.978–0.982) for the GP method, 0.980 (95% CI: 0.978–0.981) for TW3-RUS, and 0.980 (95% CI: 0.978–0.981) for expert opinion. When all three approaches were considered together, the ICC increased to 0.995 (95% CI: 0.995–0.996). As expected, average-measure ICC values were slightly higher, reaching 0.993 (95% CI: 0.993–0.994) for individual methods and 0.998 (95% CI: 0.998–0.999) when all three methods were combined. Inter-reader variability was small across assessment methods. MAD values between readers were 6.55 ± 4.91 months for the GP method, 6.58 ± 4.48 months for TW3-RUS, and 6.91 ± 4.82 months for expert opinion. When the three approaches were analyzed collectively, MAD decreased substantially to 2.55 ± 2.87 months, indicating improved agreement across readers.

**Table 2:** Inter-rater and inter-specialty reliability of bone age assessment methods

Groups	Methods	Intraclass Correlation Coefficient (95% CI)		MAD ± SD) (months)
		Single Measures ICC (3,1)	Average Measures ICC (3,k)	
All evaluators	GP	0.980 (0.978–0.982)	0.993 (0.993–0.994) ^a^	6.55±4.91
	TW3-RUS	0.980 (0.978–0.981)	0.993 (0.993–0.994) ^a^	6.58±4.48
	Expert opinion	0.980 (0.978–0.981)	0.993 (0.993–0.994) ^a^	6.91±4.82
	GP, TW3-RUS and expert opinion	0.995 (0.995–0.996)	0.998 (0.998–0.999) ^a^	2.55±2.87
Inter-specialty	GP	0.984 (0.983–0.986)	0.992 (0.991–0.993) ^b^	6.17±5.62
	TW3-RUS	0.984 (0.983–0.986)	0.992 (0.991–0.993) ^b^	5.71±5.16
	Expert opinion	0.983 (0.982–0.985)	0.992 (0.991–0.992) ^b^	6.14±5.90
	GP and TW3-RUS	0.993 (0.992–0.993)	0.996 (0.996–0.997) ^b^	3.46±4.21

CI, confidence interval; ICC, intraclass correlation coefficient; MAD, mean absolute differences; SD, standard deviation; GP, Greulich and Pyle; TW3-RUS, Tanner and Whitehouse3-Radius-Ulnar-Short bones (TW3-RUS); k, number of raters averaged in the measurement. All ICC values were statistically significant (p-value < 0.001). a = ICC (3,3), b = ICC (3,2)

Similarly, inter-specialty reliability between pediatric radiologists and pediatric endocrinologists remained high across all methods. Single-measure ICC values were 0.984 (95% CI: 0.983–0.986) for GP, 0.984 (95% CI: 0.983–0.986) for TW3-RUS, and 0.983 (95% CI: 0.982–0.985) for expert opinion. Average-measure ICC values ranged from 0.992 to 0.996, indicating consistently high agreement between specialties. MAD values between specialties were also low, measuring 6.17 ± 5.62 months for GP, 5.71 ± 5.16 months for TW3-RUS, and 6.14 ± 5.90 months for expert opinion. When GP and TW3-RUS were analyzed together, MAD decreased to 3.46 ± 4.21 months, reflecting improved agreement between specialties.

Strong correlations were observed between the two specialist groups across all three approaches, and bone age estimates derived from the GP and TW3-RUS methods were also highly correlated (*r* > 0.90, *p* < 0.01) with similar results observed using Spearman correlation.

### Comparison of mean hand bone age between pediatric endocrinologists and pediatric radiologists by assessment method

Mean bone age differences between pediatric endocrinologists and pediatric radiologists across the three assessment methods are shown in Table 3. Overall, the TW3-RUS method demonstrated a small but statistically significant inter-specialty difference (mean difference 0.686 months, *p* < 0.001), whereas GP and expert opinion showed no significant differences. This pattern was consistent in sex-stratified analyses, with TW3-RUS demonstrating significant differences in both males (*p* = 0.016) and females (*p* = 0.005).

**Table 3:** Comparison of mean bone age differences (months) between pediatric endocrinologists and pediatric radiologists assessed by each assessment method

		Paired Differences				t	df	t-test *p*-value	Non parametric *p*-value
	Mean Differences	SD of Differences	SE of Mean Differences	95% Confidence Interval of the Difference					
				Lower	Upper				
**Overall**									
Between Endo and Rad using TW3-RUS	0.686	7.670	0.185	0.322	1.049	3.697	1710	<0.001	0.011 ^a^
Between Endo and Rad using GP	0.173	8.347	0.201	-0.221	0.567	0.861	1723	0.389	0.812 ^a^
Between Endo and Rad using expert opinion	0.202	8.510	0.205	-0.200	0.604	0.983	1723	0.326	0.952 ^a^
Comparison between TW3-RUS and GP	-0.498	5.425	0.131	-0.755	-0.241	-3.804	1714	>0.001	0.315 ^a^
Comparison across TW3-RUS, GP and expert opinion	♣ Mauchly’s W = 0.441 (*p-value* = <0.001); Greenhouse-Geisser F = 25.565 (*p* = <0.001)								<0.001^b^
**Males**									
Between Endo and Rad using TW3-RUS	0.683	8.004	0.284	0.127	1.240	2.410	796	0.016	0.270 ^a^
Between Endo and Rad using GP	-0.114	8.515	0.300	-0.703	0.475	-0.379	804	0.705	0.201 ^a^
Between Endo and Rad using expert opinion	-0.010	8.351	0.294	-0.588	0.568	-0.034	804	0.973	0.162 ^a^
Comparison between TW3-RUS and GP	-0.137	5.595	0.198	-0.525	0.252	-0.690	798	0.490	0.034 ^a^
Comparison across TW3-RUS, GP and expert opinion	♣ Mauchly’s W = 0.359 (*p-value* = <0.001); Greenhouse-Geisser F = 5.879 (*p =* 0.001)								<0.001^b^
**Females**									
Between Endo and Rad using TW3-RUS	0.688	7.370	0.244	0.209	1.166	2.821	913	0.005	0.014 ^a^
Between Endo and Rad using GP	0.424	8.193	0.270	-0.106	0.955	1.570	918	0.117	0.136 ^a^
Between Endo and Rad using expert opinion	0.387	8.647	0.285	-0.173	0.947	1.356	918	0.175	0.159 ^a^
Comparison between TW3-RUS and GP	-0.814	5.255	0.174	-1.155	-0.473	-4.687	915	<0.001	0.001 ^a^
Comparison across TW3-RUS, GP and expert opinion	♣ Mauchly’s W = 0.513 (*p-value* = <0.001); Greenhouse-Geisser F = 25.482 (*p* = <0.001)								<0.001^b^

*SD*, standard deviation; *SE*, standard error; *CI*, confidence interval; *t*, t statistic; *df*, degrees of freedom; *Endo*, pediatric endocrinologists; *Rad*, pediatric radiologists; *TW3-RUS*, Tanner and Whitehouse 3 radius-ulnar-short bones; *GP*, Greulich and Pyle; statistical significance for a *p*-value < 0.05. a = using Wilcoxon Signed Ranks test, b = using Friedman’s test

**Fig. 3:**
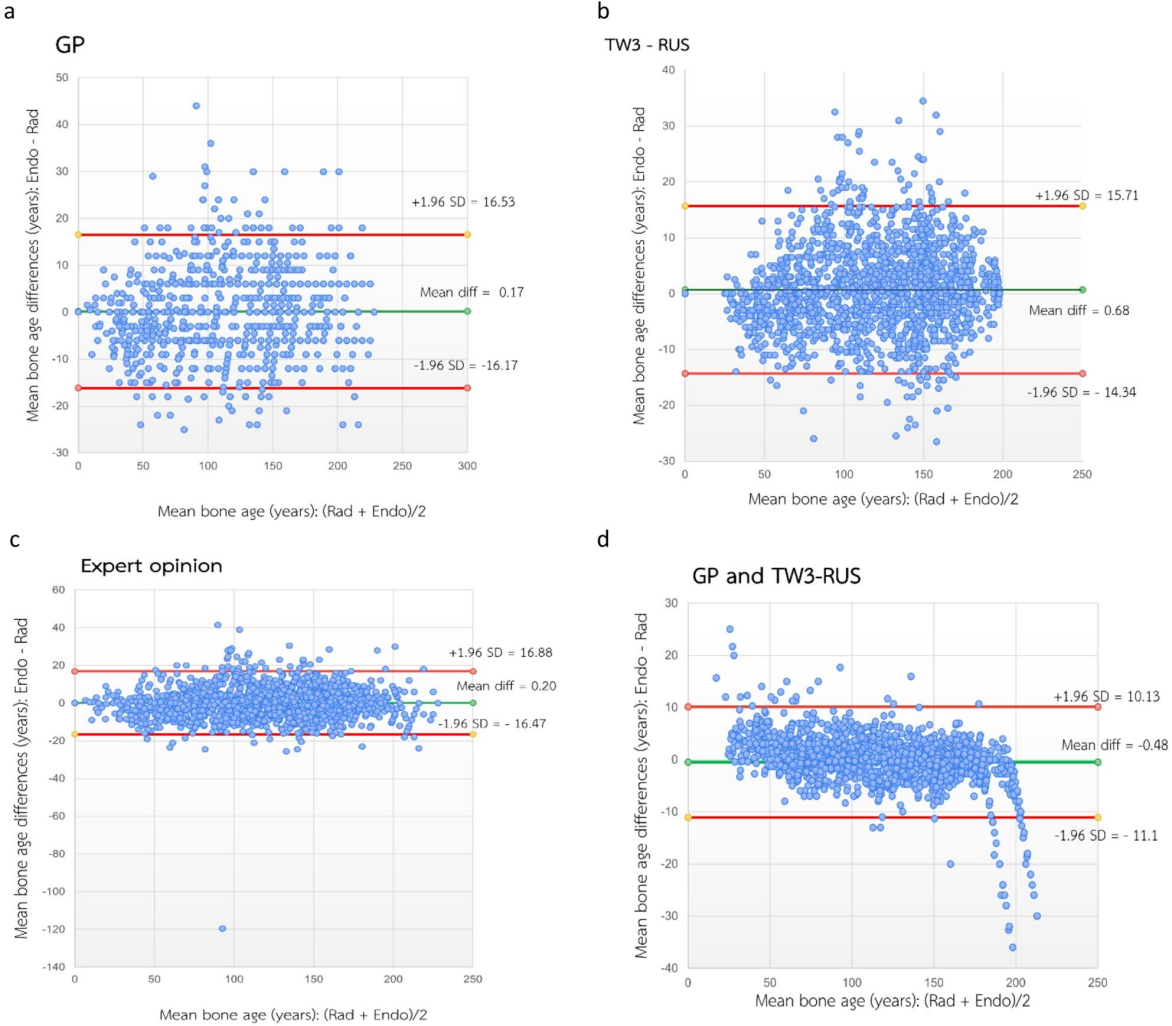
Bland-Altman plots for the mean difference of bone ages assessed by two specialist groups using each method. (**a**) GP method (**b**) TW3-RUS method (**c**) Expert opinion (**d**) GP and TW3-RUS methods

Bland–Altman analysis further demonstrated a high level of agreement between pediatric radiologists and endocrinologists across the three BAA approaches (Fig. 3). Mean differences were 0.17 months for GP (95% limits of agreement [LoA], − 16.17 to 16.53), 0.68 months for TW3-RUS (95% LoA, − 14.34 to 15.71), and 0.20 months for expert opinion (95% LoA, − 16.47 to 16.88). When GP and TW3-RUS were analyzed jointly, the mean difference was − 0.48 months with narrower LoA (− 11.10 to 10.13), indicating improved agreement between specialties

Direct comparison between assessment methods showed that TW3-RUS produced slightly lower bone age estimates than GP overall (mean difference − 0.498 months, *p* < 0.001), a difference that was particularly evident in females. Repeated-measures analysis confirmed significant differences among TW3-RUS, GP, and expert opinion methods overall and in both sex groups (*p* < 0.001).

Wilcoxon signed-rank tests were performed as a sensitive analysis and yielded results largely consistent with those of the paired *t*-tests. Minor discrepancies were observed. The overall difference between TW3-RUS and GP was statistically significant with the paired *t*-tests but not within the Wilcoxon signed-rank test (*p* = 0.315). Similarly, the difference between radiologists and endocrinologists using TW3-RUS method in males was significant by *t*-test but not by Wilcoxon test (*p* = 0.270). In contrast, for the comparison between TW3-RUS and GP in males, the Wilcoxon signed-ranks test indicated a significance difference (*p* = 0.034), whereas the paired *t*-test did not (*p* = 0.490). For comparisons among the three assessment methods (TW3-RUS, GP, and expert opinion), results from the Greenhouse–Geisser corrected repeated-measures ANOVA were consistent with those obtained using the non-parametric Friedman test. Overall, the main conclusions remained consistent across both parametric and non-parametric statistical approaches.

### Age-stratified agreement by assessment method

Across most age groups, mean bone age estimates obtained using the GP method, TW3-RUS method, and expert opinion were generally comparable to chronological age in both sexes. In younger age groups (0–36 and 36.1–84 months), TW3-RUS tended to yield higher bone age estimates than GP and expert opinion in both males and females. In contrast, in older age groups, particularly after 180 months, TW3-RUS tended to produce lower estimates compared with GP and expert opinion (Table 4).

**Table 4:** Chronological age and mean bone age assessed by expert readers, stratified by sex and assessment method

Chronological Age groups	No.		Chronological age (months)				Bone Age (months)											
							GP				TW3-RUS				Expert opinion			
	Male	Female	Male		Female		Male		Female		Male		Female		Male		Female	
			**Mean**	**SD**	**Mean**	**SD**	**Mean**	**SD**	**Mean**	**SD**	**Mean**	**SD**	**Mean**	**SD**	**Mean**	**SD**	**Mean**	**SD**
0–36	18	27	26.48	6.90	22.04	8.70	30.56	18.87	30.44	14.82	43.51	13.78	37.45	14.28	30.24	18.53	32.02	16.09
36.1–84	167	173	61.14	13.21	67.47	13.48	64.76	33.23	73.85	24.28	66.80	32.10	74.60	23.03	65.54	32.84	74.03	23.79
84.1–132	238	445	108.69	13.88	105.43	13.60	113.27	37.25	119.47	23.92	114.42	36.13	119.10	23.78	113.80	37.08	119.54	23.92
132.1–180	260	210	154.68	13.46	151.41	13.62	151.67	30.47	151.79	22.15	150.97	28.94	150.50	20.52	151.84	30.29	151.76	21.89
180.1–216	116	59	193.68	9.88	193.99	10.40	182.20	29.45	178.29	22.55	177.32	23.57	170.11	14.67	182.29	28.85	178.46	21.93
≥216.1	6	6	223.61	3.02	224.24	6.95	172.00	54.66	182.33	25.56	165.50	48.43	170.40	9.54	171.27	54.31	182.35	25.59

No., number; GP, Greulich and Pyle; TW3-RUS, Tanner and Whitehouse3-Radius-Ulnar-Short bones (TW3-RUS); SD, standard deviation

The MAD in bone age assessment between the two specialties and across methods are presented in Table 5. Overall, MAD values between specialties were relatively small across most age groups and methods, generally ranging from approximately 4 to 7 months. Across age groups, the TW3-RUS method generally demonstrated comparable or slightly lower MAD values than the GP method in both males and females.

**Table 5:** Mean absolute differences (MAD) in bone age assessments between two specialties and methods, stratified by sex and chronological age group

Chronological Age groups	No.		Mean Absolute Differences (months)															
			MAD_GP				MAD_TW3-RUS				MAD_Expert opinion				MAD_GP vs. TW3-RUS			
	Male	Female	Male		Female		Male		Female		Male		Female		Male		Female	
			**Mean**	**SD**	**Mean**	**SD**	**Mean**	**SD**	**Mean**	**SD**	**Mean**	**SD**	**Mean**	**SD**	**Mean**	**SD**	**Mean**	**SD**
0–36	18	27	5.89	4.38	4.15	4.12	5.05	4.89	2.67	2.18	5.14	4.52	3.79	3.26	6.48	7.81	5.33	4.16
36.1–84	167	173	5.56	5.17	5.63	5.49	5.08	3.99	5.28	4.47	5.89	4.78	5.30	4.90	3.25	2.84	2.81	2.70
84.1–132	238	445	6.93	5.93	6.34	5.70	6.34	5.54	6.33	4.84	6.76	5.81	6.54	7.32	2.89	2.49	2.58	2.02
132.1–180	260	210	6.72	5.92	5.67	5.25	6.60	6.09	5.29	4.88	6.34	5.71	5.53	4.75	2.84	3.20	3.13	3.21
180.1–216	116	59	5.56	5.41	6.99	6.28	4.27	5.80	3.49	4.92	5.90	5.61	6.96	5.98	6.82	7.51	9.32	9.97
≥216.1	6	6	5.00	4.10	4.50	3.67	3.75	4.01	5.00	5.81	5.42	3.07	4.92	4.20	7.50	7.44	12.83	16.48

No., number; MAD_GP, mean absolute difference in bone age between two specialties using Greulich and Pyle (GP) method; MAD_TW3-RUS mean absolute difference in bone age between two specialties using Tanner and Whitehouse3-Radius-Ulnar-Short bones (TW3-RUS) method; MAD_expert opinion, mean absolute difference in bone age between two specialties based on expert opinion; MAD_GP vs. TW3-RUS, mean absolute difference between GP and TW3-RUS across all evaluators; SD, standard deviation

Across age groups, MAD values were slightly higher in the 84.1–180 months group when a single method was used, whereas smaller differences were observed in younger children. When comparing GP and TW3-RUS directly, MAD values were generally modest in most age groups (approximately 2.5–3.3 months) but increased in older adolescents, particularly in the 180.1–216 months and ≥216.1 months groups. These findings indicate that variability between specialties and between assessment methods was generally limited, although somewhat greater differences were observed near skeletal maturity.

### Consistency of repeated ratings by the same evaluator

Intra-rater reliability between repeated reading rounds was excellent across all BAA methods (Table 6). For the GP method, the single-measure ICC was 0.974 (95% CI: 0.971–0.976) between rounds 1 and 2 and 0.955 (95% CI: 0.939–0.967) between rounds 2 and 3. For the TW3-RUS method, the corresponding ICC values were 0.967 (95% CI: 0.964–0.971) and 0.958 (95% CI: 0.942–0.969). Expert opinion demonstrated similarly high agreement, with ICC values of 0.971 (95% CI: 0.967–0.974) between rounds 1 and 2 and 0.912 (95% CI: 0.881–0.935) between rounds 2 and 3. Average-measure ICC values ranged from 0.954 to 0.987, reflecting improved reliability when repeated readings were averaged across raters.

**Table 6:** Intra-rater reliability of individual readers stratified by bone age assessment method

Methods	Round Comparison	Intraclass Correlation Coefficient (95%CI)		MAD ± SD) (months)
		Single Measures model ICC (3,1)	Average Measures model ICC (3,2)	
GP	R1 vs. R2	0.974 (0.971–0.976)	0.987 (0.985–0.988)	6.26±7.45
	R2 vs. R3	0.955 (0.939–0.967)	0.977 (0.968–0.983)	5.99±7.11
TW3-RUS	R1 vs. R2	0.967 (0.964–0.971)	0.983 (0.982–0.985)	7.22±7.36
	R2 vs. R3	0.958 (0.942–0.969)	0.978 (0.970–0.984)	6.94±5.52
Expert opinion	R1 vs. R2	0.971 (0.967–0.974)	0.985 (0.983–0.987)	6.51±7.91
	R2 vs. R3	0.912 (0.881–0.935)	0.954 (0.937–0.967)	7.76±10.42

CI, confidence interval; ICC, intraclass correlation coefficient; MAD, mean absolute differences; SD, standard deviation; GP, Greulich and Pyle; TW3-RUS, Tanner and Whitehouse3-Radius-Ulnar-Short bones; R1, first round of independent assessment; R2, second round; R3, third round. All ICC values were statistically significant (*p* < 0.001)

The magnitude of intra-reader differences was modest, with mean absolute differences (MAD) ranging from 5.99 to 7.76 months across methods and reading intervals.

### Impact of reader experience on agreement in bone age assessment

MAD values were broadly comparable across experience levels, with mean ± SD of 3.84 ± 3.30 months (< 6 years), 4.01 ± 3.43 months (6–9 years), and 4.15 ± 3.50 months (> 9 years), and an overall mean of 4.02 ± 3.43 months. Median MAD values were similar across groups (3.00-3.33 months). The ranges were 0–24.00 months for both the < 6 and 6–9 year groups and 0–27.67 months for > 9 year group (overall range, 0–27.67 months).

Normality testing indicated a non-normal distribution (*p* < 0.001); however, ANOVA demonstrated a statistically significant difference among groups (F = 4.344, *p* = 0.013) with no violation of homogeneity of variances (Levene’s *p* = 0.058). Post hoc analysis showed overlapping subsets, indicating no clear pairwise differences. These findings were supported by the Kruskal–Wallis test (χ² = 7.752, *p* = 0.021). Overall, the differences in MAD were small.

### Duration required for BAA by pediatric radiologists and pediatric endocrinologists using each method

Pediatric radiologists required less time to complete assessments than pediatric endocrinologists for both the GP and TW3-RUS methods. The mean time required for bone age interpretation using the GP method by pediatric radiologists and pediatric endocrinologists was 1.33 min (SD 0.52) and 1.42 min (SD 0.80), respectively. For the TW3-RUS method, the mean time was 3.83 min (SD 1.33) and 5.33 min (SD 2.94), respectively. In addition, the overall mean time for BAA by both expert groups was 1.38 min (SD 0.64) with the GP method, and 4.58 min (SD 2.31) with the TW3-RUS method (*p* = 0.002).

### Expert perspectives on Greulich–Pyle (GP) versus Tanner–Whitehouse 3 (TW3) methods for bone age assessment

#### Usability and efficiency (speed and workflow)

All experts (100%) agreed that GP was faster, easier to use, and reduced workload in general practice. Regarding diagnostic performance, 58.3% rated TW3-RUS as more accurate and 66.7% as more specific. In term of sensitivity, 41.7% favored TW3-RUS, whereas 33.3% favored GP. However, some experts agreed that both methods demonstrated comparable accuracy (41.7%), specificity (16.7%) and sensitivity (25.0%) in this study, as the lack of true chronological age data limited direct comparison.

#### Limitations and pitfalls

Experts reported that GP was limited by widely spaced age intervals in the atlas and by skeletal variability that could introduce interpretation errors (66.7%). They also reported that TW3 had limited applicability for bone age below 2 years or above 15–16 years and generally required more time to evaluate. For readers with limited reader experience, the use of the TW3 method may be challenging. Although descriptive criteria for scoring each bone are provided, the relatively small bone images in the atlas may lead to scoring inaccuracies for individual bones.

#### Education and clinical utilization

Half of the experts (50.0%) preferred GP for teaching, citing greater ease of understanding and suitability for beginners. For routine clinical use, 75.0% considered GP more appropriate because it is faster and simpler to apply. Regarding treatment planning, 41.7% reported no substantial difference between the methods; nevertheless, TW3 was noted to provide greater granularity and was favored for complex cases. When GP results were inconclusive or patients fell into ambiguous age ranges, experts preferred to use TW3 to support decision-making.

#### Future directions

Experts indicated that both methods could be advanced to AI-based assessment; they considered GP more amenable to clinical automation, with TW3 serving as an adjunct where higher precision was required.

#### Final recommendation

Experts generally considered GP preferable for routine clinical use due to its speed and ease, whereas TW3 was preferred when more in-depth information or complex diagnostic scenarios were involved; some recommended using both methods together to leverage strengths and mitigate limitations. Furthermore, the development of a method analogous to GP, but with narrower or more finely defined age intervals, could offer a promising alternative for clinical application.

## Discussion

In this large, cross-specialty study of pediatric hand radiograph BAA, we observed excellent agreement both within and between pediatric radiologists and endocrinologists across all approaches—GP, TW3-RUS, and an expert opinion. Interrater reliability was consistently high (ICC > 0.9 for all approaches), and intra-rater reliability demonstrated strong stability across repeated readings, indicating excellent repeatability. Correlations between assessment methods and between specialties exceeded 0.90, further supporting the strong concordance across evaluators. In addition, MAD values between readers and specialties were low across methods with aggregated averages of approximately 5–6 months for GP and about 5 months for TW3-RUS, indicating minimal variability. These findings align with previous reports demonstrating high inter-observer reliability and strong reproducibility for both the GP and TW3 methods among trained readers [10–15], as well as excellent intra-observer reliability for both methods [10, 16–18]. For GP-based assessments, this level of variability is also comparable to that reported for automated and deep learning–based systems [19–21]. Although Bland-Altman analysis revealed minimal systematic bias between specialties, single-method assessments showed relatively wide LoA (e.g., GP *±* 16 months), indicating that individual patient-level differences may occur despite strong overall concordance. Notably, integrating GP and TW3-RUS narrowed the LoA to approximate *±* 10 months, suggesting improved interobserver consistency when both approaches are considered together.

Inter-specialty differences varied by assessment method. TW3-RUS demonstrated a statistically significant difference between specialties, whereas GP and expert opinion did not. However, the absolute magnitude of these differences was small and unlikely to be clinically meaningful in most cases. Specialty-related variation using TW3-RUS was observed in both sexes and was slightly more evident among females, although the magnitude of the difference remained modest. This pattern may relate to the accelerated tempo of skeletal maturation during puberty, particularly in females, where rapid progression through epiphyseal stages may increase sensitivity to minor inter-reader differences. Direct comparison between methods showed TW3-RUS tended to yield slightly lower bone age estimates than GP, consistent with prior reports [22]. These differences likely reflect calibration differences between scoring systems rather than true biological discrepancies.

Across age groups, the TW3-RUS method generally demonstrated comparable or slightly lower MAD values than the GP method. This may reflect the structured scoring framework of TW3-RUS, which evaluates specific ossification stages of multiple hand bones and converts standardized scores into bone age estimates. Such a quantitative approach may reduce subjective interpretation compared with the atlas-based GP method, which relies more heavily on visual comparison with reference images. Sensitivity analyses using Wilcoxon signed-rank tests produced results largely consistent with the paired *t*-tests, supporting the robustness of the findings despite minor deviations from normality. Although a few comparisons differed in statistical significance between the two approaches, the overall pattern of results remained unchanged.

While the mean differences between methods were modest, the observed LoA indicate that individual-level variability may occasionally exceed one year. This dispersion reflects expected variation between assessment approaches rather than systematic bias. In routine clinical practice, such differences are unlikely to affect management in most patients but may become relevant when bone age estimates approach diagnostic or therapeutic thresholds. Greater variability in bone age estimates was observed in the 84.1–180 months age groups, where MAD values were slightly higher than in younger children. This period corresponds to mid-childhood and early adolescence, during which skeletal maturation progresses through transitional stages and subtle epiphyseal changes may be interpreted differently by readers, particularly when ossification centers approach key developmental milestones. A previous study has similarly reduced reliability of bone age assessment in 9–14 years using GP atlas [23]. When comparing GP and TW3-RUS directly, MAD values were generally modest across most age groups (approximately 2.5–3.3 months), indicating good agreement between methods. However slightly larger differences were observed in older adolescents (> 180 months). Near skeletal maturity, many epiphyses are either fusing or have already fused, which reduces the number of remaining developmental markers available for age estimation. This limited morphological variation may increase the sensitivity of bone age estimation to small interpretative differences or methodological calibration differences between scoring systems. Despite these variations, the overall agreement between specialties and assessment methods remained strong across all age groups, suggesting that both approaches provide reliable bone age estimates in clinical practice.

MAD values were comparable across experience levels, with similar mean and median values, indicating minimal impact of reader experience on agreement in bone age assessment. The close alignment of median MAD values (3.00-3.33 months) suggests a stable central tendency across groups. Although statistically significant differences were observed, post hoc analysis showed no clear pairwise differences, and the absolute differences were small, indicating limited clinical relevance. The wide MAD range (0-27.67 months) likely reflect occasional outliers rather than systemic variability, possibly related to more challenging cases near skeletal maturity. These relatively small differences across experience levels may reflect the effect of standardized training and the structured nature of bone age assessment methods, which help reduce inter-reader variability.

From a practical perspective, the two methods differ in efficiency. GP was substantially faster than TW3-RUS (mean 1.4 vs. 4.6 min for TW3-RUS) and generally preferred for routine use due to its intuitive atlas approach. Although reading times are slightly higher than reported by Yuh et al. (0.79 min for GP; 3.01 min for TW3-RUS) [12], the same pattern of GP’s efficiency persisted. Although this difference reached statistical significance, the absolute per-case time difference—less than five minutes overall—may have limited clinical impact for individual assessments. In high-volume clinical settings, however, even small efficiency gains may accumulate and translate into meaningful workflow improvements. TW3-RUS provides structured staging and greater granularity but requires longer interpretation time and has a restricted validated age range, limiting its practicality as a universal first-line method.

We selected the RUS component of TW3 rather than the carpal or combined score because it is more reliable across the clinically relevant pediatric age range, correlates more strongly with chronological age, and shows lower inter-observer variability [7, 12]. Carpal bones mature earlier and provide a narrower discriminatory window, while their inclusion increases complexity and reading time without consistent gains in accuracy for older children. Given that TW3-RUS was already slower than GP in our study, adding the carpal component would further prolong interpretation without clear benefit. Moreover, TW3’s validated range begins around two years, when RUS features are already informative, minimizing the incremental value of carpal scoring. Thus, TW3-RUS offers the most balanced combination of precision and feasibility for clinical application.

Ethnicity is an important consideration in bone age assessment. Both the GP and TW3 reference standards were originally developed using predominantly mid-20th century North American Caucasian or European populations [6, 7]. Subsequent studies have demonstrated that skeletal maturation patterns may vary across ethnic groups, with differences observed in the timing and tempo of epiphyseal development [8, 19, 24–27]. Such variations may influence absolute bone age estimates when these references are applied to non-European populations. Our cohort consisted exclusively of Thai children evaluated at a tertiary-care center. Although our primary aim was to assess inter-method agreement and inter-reader reliability rather than absolute validation against a population-specific standard, potential ethnicity-related calibration differences should be considered when interpreting the findings and generalizing them to other populations.

Artificial intelligence (AI)-based bone age assessment systems have shown promising performance in recent studies [3, 20, 28, 29]; however, they were not the focus of this study. The high level of agreement observed between pediatric radiologists and endocrinologists highlights the reliability of manual atlas-based assessment and supports its role as a robust clinical standard. While AI may serve as an adjunct to enhance standardization, reduce variability, and improve workflow efficiency—particularly in high volume settings—its application requires further validation across diverse populations and specific clinical contexts. Consequently, clinical judgment and inter-disciplinary agreement remain central to diagnostic decision-making.

A pragmatic, tiered approach is recommended for pediatric BAA, using the GP atlas as the default for routine assessments and reserving TW3-RUS for equivocal cases, complex maturational stages (e.g., peripubertal period), or when fine-grained staging is needed. For serial evaluations guiding therapy, consistency should be ensured by using a single documented method, with confirmatory readings recommended when results approach therapeutic thresholds. Although inter-specialty concordance between pediatric radiologists and endocrinologists was high, individual readings may occasionally differ by more than a year, particularly during the period of skeletal maturity or near clinical decision boundaries. Therefore, bone age estimates should always be interpreted within the full clinical context—considering growth velocity, pubertal tempo, parental heights, physical findings, and endocrine results—and repeated or alternative assessments should be pursued when findings are borderline or discordant.

This study has several strengths, including a large sample size, representation from two core clinical specialties, objective time-motion data, and rigorous assessment of inter- and intra-rater reliability. Multiple complementary statistical methods and triangulation across GP, TW3-RUS, and expert opinion provided a robust evaluation of agreement. Nevertheless, several limitations should be acknowledged. Because GP and TW3-RUS assessments were performed within the same reading session, a degree of sequential or anchoring bias cannot be entirely excluded, although each method was recorded independently. The exclusion of TW3 out-of-range cases may have reduced observed variability and subtly influence method comparisons. The absence of an external biological gold standard means that our findings address inter-method agreement rather than absolute accuracy. The study population consisted exclusively of Thai children; therefore, the findings may not be generalizable to other ethnic groups or populations with different secular growth patterns. Finally, time-motion results reflect the specific practice environment and reader experience studied here and may differ in other settings or with automated assessment tools. While mean bias was minimal, the limits of agreement indicate that clinically meaningful individual differences may occur, particularly around puberty or near therapeutic thresholds.

Future work should explore hybrid workflows in which the GP atlas serves as the default approach, with predefined criteria for escalation to TW3-RUS in cases of equivocal GP findings or when greater stage precision is required. Prospective studies are warranted to determine whether small method-related differences in estimated bone age influence treatment decisions or outcomes. Given the greater time demands of TW3-RUS, AI–assisted tools may help streamline such approaches by facilitating atlas matching, identifying ambiguous cases, and improving consistency. Beyond workflow considerations, addressing calibration bias remains an important priority. Because both GP and TW3 were derived from historical reference cohorts, the development of a population-specific, sex-stratified digital atlas of hand skeletal maturity—analogous to the GP atlas, incorporating narrower and precisely defined age intervals—may improve accuracy and generalizability. This atlas should undergo external validation and periodic recalibration to capture secular trends. Large multicenter studies across diverse populations will be essential to refine calibration and inform method selection, ensuring that hybrid diagnostic protocols are optimized for both accuracy and clinical efficiency.

In summary, pediatric radiologists and pediatric endocrinologists achieved very high agreement in hand bone age assessment using both GP and TW3-RUS. GP was substantially faster and preferred for routine practice, whereas TW3-RUS, while slower and age-limited, offered greater granularity and produced slightly lower age estimates than GP. These findings support a pragmatic, complementary strategy: use GP as the first-line method, apply TW3-RUS selectively in complex or borderline cases.

## Data Availability

Summary data supporting the findings of this study are presented within the article. Further inquiries can be directed to the corresponding author.
